# Frequency of low ImPACT scores among adolescent and young adult student-athletes with attention-deficit/hyperactivity disorder and/or learning disorder

**DOI:** 10.3389/fneur.2024.1446962

**Published:** 2024-11-19

**Authors:** Philip Schatz, John Cuzzupe, Justin E. Karr, Nathan E. Cook, Grant L. Iverson

**Affiliations:** ^1^Department of Psychology, Saint Joseph’s University, Philadelphia, PA, United States; ^2^Department of Psychology, University of Kentucky, Lexington, KY, United States; ^3^Department of Physical Medicine and Rehabilitation, Harvard Medical School, Boston, MA, United States; ^4^Department of Physical Medicine and Rehabilitation, Spaulding Rehabilitation Hospital, Charlestown, MA, United States; ^5^Mass General for Children Sports Concussion Program, Waltham, MA, United States; ^6^Department of Physical Medicine and Rehabilitation, Schoen Adams Research Institute at Spaulding Rehabilitation, Charlestown, MA, United States

**Keywords:** concussion, multivariate base rates, neurocognitive, impact, attention-deficit/hyperactivity disorder, learning disorder

## Abstract

**Introduction:**

Attention-deficit/hyperactivity disorder (ADHD) is a neurodevelopmental disorder characterized by attention problems, excessive activity, and impulsivity - occurring in approximately 13% of children 12–17 years of age, and as many as 16% of older adolescents 18–22 years of age, with a greater prevalence in boys than girls. ADHD frequently co-occurs with specific learning disorder (LD), a neurodevelopmental disorder characterized by difficulties learning and using academic skills, such as reading, writing, and mathematics. Taken together, ADHD and/or LD are common among high school students and college students and can influence performance on computerized tests used in concussion management. However, normative data for widely used computer-based measures used in concussion management do not include reference samples with ADHD and/or LD. Previous research has documented the expected frequency of obtaining low scores on computer-based neuropsychological test measures among healthy, uninjured individuals, but few studies have examined the frequency of obtaining low scores in athletes with neurodevelopmental diagnoses, such as ADHD and/or LD. This study examined the frequency of low scores (i.e., multivariate base rates) on the Immediate Post-Concussion Assessment and Cognitive Test (ImPACT) for youth and young adults with self-reported ADHD and/or LD.

**Methods:**

The sample included 174,878 adolescent and young adult student-athletes who completed pre-season baseline neurocognitive assessments, who were assigned to the following independent groups: ADHD only (*n* = 45,215), LD only (*n* = 54,223), ADHD and LD (*n* = 45,737), No ADHD or LD (*n* = 47,684; “control athletes”).

**Results:**

Base rates of low scores were stratified by high school (e.g., 13–18) and collegiate age (e.g., 19–22) and sex. It was common for student athletes (all ages) with LD alone (i.e., 30–37%), or with both ADHD and LD (i.e., 24–31%), to obtain at least two low ImPACT composite scores, but not three low composite scores. However, it was relatively uncommon for control athletes (those without ADHD or LD) (i.e., 12–14%) or older athletes (ages 19–22) with ADHD (i.e., 14–15%) to obtain two (or more) low scores.

**Discussion:**

Having information relating to the base rates of low scores among uninjured athletes enhances the interpretation of ImPACT results among the broader population of student-athletes with and without neurodevelopmental disorders.

## 1 Introduction

Neurocognitive testing is commonly used in concussion management (1, 2), and the Immediate Post-Concussion Assessment and Cognitive Testing (ImPACT) battery is among the most frequently used cognitive assessment batteries for this purpose (3). There are two primary ways to interpret ImPACT results when the battery is administered following a concussion or suspected concussion. If the student-athlete underwent baseline or preparticipation testing, then post-injury test performances can be compared to baseline (pre-injury) performance (4). Alternatively, post-injury performances can be compared to normative reference data (5), or results from a group of uninjured athletes who are similar to the examinee, such as being the same sex and similar age. Clinicians could also apply both interpretive approaches (6).

The interpretation of neurocognitive test battery results requires consideration of normal variability in test performance, or the frequency at which healthy, uninjured individuals obtain scores that could be considered “low” or “abnormal.” Numerous studies over the past 15 years have documented that normal, healthy children, adolescents, adults, and older adults often obtain low scores when administered a battery of neuropsychological tests (7–12). This finding has been quantified by calculating multivariate base rates, which refer to the proportion of healthy participants in normative samples who obtain one or more scores falling below a certain threshold or distance from the mean (e.g., <16th percentile, ≤5th percentile, etc.). Such multivariate base rate information is important to promote accurate interpretation of performance across a test battery, such as ImPACT, which includes four primary composite scores. A given athlete might obtain a score on one composite that, in isolation, falls far enough below the normative mean as to suggest potential clinically significant difficulty. However, multivariate base rate information is necessary to interpret isolated low scores more fully, because, as noted above, dozens of studies have shown that obtaining one or more low scores is common among uninjured individuals when multiple tests are administered. Therefore, concluding that isolated low scores represent impairment or dysfunction that might be associated with a sport-related concussion, absent multivariable base rate information, raises the risk for a potential false positive diagnostic error or inaccurate clinical conclusion (if, for example, a relatively large proportion of uninjured individuals also obtain that number of low scores).

Prior research on the ImPACT battery has found that, consistent with the broader literature on other neurocognitive batteries referenced above, it is relatively common for uninjured adolescents and young adults to obtain low scores across the four composites (8, 13). For example, two out of five uninjured high school and collegiate athletes (about 40%) obtain at least one of the four ImPACT composite scores at or below the 16th percentile (one standard deviation below the normative mean) and between one in six and one in seven (roughly 15%) obtain two or more composites at or below the 16th percentile. A hypothetical case example from Iverson and Schatz (8) illustrates the clinical utility of multivariate base rate information. A 20-year-old young woman obtained one score below the 10th percentile when she was tested before the season (i.e., at baseline), a result that occurs in approximately 28% of young women her age. Following a concussion or suspected concussion she was retested and obtained three scores below the 10th percentile, a result that occurs in fewer than 2% of women her age. Thus, her post-injury test results, when interpreted using multivariate base rates of low scores, convincingly suggest diminished performance relative to her preseason baseline and also normative expectations.

In addition to multivariate base rate information, the interpretation of neurocognitive test scores also requires consideration of factors that might be associated with lower test performance, such as pre-existing conditions and neurodevelopmental disorders. Attention-deficit/hyperactivity disorder (ADHD) is a neurodevelopmental disorder characterized by attention problems, excessive activity, and impulsivity (13). ADHD occurs in approximately 10% of children 6–11 years of age and 13% of children 12–17 years of age, with a greater prevalence in boys (13%) than girls (6%) (14). Prevalence of ADHD in college-age students ranges from 6% (15) to 16% (16). ADHD frequently co-occurs with specific learning disorder (LD), a neurodevelopmental disorder characterized by difficulties learning and using academic skills, such as reading, writing, and mathematics (17). Some estimates suggest that up to 60% of youth with ADHD also meet criteria for an LD (18, 19). Both of these neurodevelopmental conditions have been associated with differences in ImPACT performance. On average, youth with ADHD (20–23) and youth with LD (23–26) obtain lower scores on ImPACT than youth who do not have these conditions.

Taken together, prior research has reported multivariate base rates of low scores among uninjured high school and collegiate athletes on ImPACT and a separate literature indicates that uninjured athletes with ADHD and LD, on average, obtain lower ImPACT scores than athletes without these neurodevelopmental conditions. However, the base rates of low scores among uninjured athletes with ADHD and/or LD have not been reported. Thus, the purpose of this study was to calculate the frequency of obtaining low scores during preseason baseline ImPACT testing for adolescent and young adult student-athletes with ADHD and LD.

## 2 Materials and methods

### 2.1 Participants

Deidentified data were obtained from ImPACT Applications, Inc. The initial sample consisted of 187,625 uninjured student-athletes who completed baseline, preparticipation ImPACT. First, high school student-athletes (*n* = 145,947; 95,686 males, 50,261 females) and collegiate student-athletes (*n* = 17,096; 10,984 males, 6,112 females) with self-reported ADHD and/or LD (note: LD included athletes with self-reported a history of learning disorder or dyslexia) who completed the online version ImPACT (Version 4) were selected. Second, a group of student-athletes without ADHD or LD (*n* = 49,540; 23,896 males, 25,644 females) was included as a comparison/control group. All ImPACT administrations were completed in English and obtained from organizations within the United States. If more than one baseline assessment was available for a given student-athlete, only the first baseline was included. Student-athletes were excluded if they reported sustaining a concussion within the 6 months prior to their baseline assessment or if they reported a history of brain surgery, meningitis, epilepsy/seizures, and/or a treatment for substance or alcohol abuse. ImPACT has embedded validity indicators (EVIs), which identity “invalid” or “sub-optimal” performance on baseline assessments by identifying performance below the 5th percentile as compared to normative data stratified by age and sex (27). Baseline results identified as “invalid” (as denoted by “Baseline ++” per the ImPACT embedded validity indicators) were removed (ADHD only = 8.3%, LD only =12.0%, ADHD and LD = 12.3%, No ADHD or LD = 3.7%), yielding the following final study groups: ADHD only (*n* = 45,215) LD only (*n* = 54,223), ADHD and LD (*n* = 45,737), No ADHD or LD (*n* = 47,684). Demographic characteristics of the sample and subgroups are provided in Table 1. The standard normative reference values for each ImPACT test score were used for this study.

**Table 1:** Demographic characteristics of the sample.

	ADHD only	LD only	ADHD + LD	No ADHD or LD (controls)
	(*n* = 45,215)	(*n* = 54,223)	(*n* = 45,737)	(*n* = 47,684)
Age, mean ± SD	15.79 ± 2.00	15.86 ± 2.01	16.03 ± 2.07	16.66 ± 1.92
Sex				
Male, *n* (%)	32,047 (71%)	32,672 (60%)	29,865 (65%)	22,875 (48%)
Female, *n* (%)	13,168 (29%)	21,551 (40%)	15,872 (35%)	24,809(52%)
Prior concussion*				
None, *n* (%)	32,388 (75.6%)	40,335 (79.4%)	31,244 (72.3%)	37,167 (81.6%)
One, *n* (%)	6,498 (15.2%)	6,767 (13.3%)	7,310 (16.9%)	5,801 (12.7%)
Two+, *n* (%)	3,933 (9.2%)	3,714 (7.3%)	4,671 (10.8%)	2,573 (5.6%)

*Prior concussion data were missing for 5.6% of the sample; ADHD, Attention-deficit/hyperactivity disorder; LD, Learning disability. Prior concussion data were missing by subgroup as follows: ADHD Only = 5.3%, LD Only = 6.3%, ADHD + LD = 5.5%, and No ADHD or LD (controls) = 4.5%.

### 2.2 Measures

ImPACT is a computerized test battery that assesses neurocognitive functioning and post-concussion symptoms. Athletes first complete a section where they self-report demographic data including several aspects of their health history (including whether they have ADHD and LD). Next, athletes complete the Post-Concussion Symptom Scale (PCSS). Last, athletes complete several neurocognitive tests measuring attention, memory, processing speed, and reaction time (27), and various scores derived from those tests are combined into four composite scores: Visual Memory, Verbal Memory, Visual Motor Speed, and Reaction Time. ImPACT has been shown to have moderate-to-high levels of sensitivity and specificity for detecting change following diagnosed concussion (28, 29). However, the reliability data are mixed, with some studies indicting low reliability (30, 31), others indicating moderate reliability, and still others suggesting high test–retest reliability (32–34).

### 2.3 Procedures

Data were exported by ImPACT Applications Inc. for institutions that provided consent for their de-identified data to be used for research purposes. Self-reported diagnoses of ADHD and LD were identified based on each athlete’s response to questions in the demographic/health history section of ImPACT. Based on responses to the ImPACT demographic questionnaire, student-athletes were assigned to the following four independent groups: ADHD Only, LD Only, ADHD and LD (ADHD+LD), and neither ADHD nor LD (No ADHD or LD). ImPACT composite scores can be transformed into percentile ranks comparing an individual’s performance to age-referenced normative data (i.e., based on single year age bands for youth ages 12–18 and larger age bands for older individuals) stratified by sex (27). The total standardization sample for ImPACT version 4 consisted of 72,369 individuals who completed baseline ImPACT testing and excluded individuals who self-reported ADHD or LD (27). Composite raw scores for the current study sample (Verbal Memory, Visual Memory, Motor Speed, Reaction Time) were converted to percentile ranks using ImPACT normative values. These are the same standard normative reference values that are provided by the test publisher.

### 2.4 Statistical analyses

Analyses of variance (ANOVAs) were used to compare ADHD, LD, and ADHD+LD groups on the four ImPACT neurocognitive composite raw scores: Verbal Memory, Visual Memory, Motor Speed, and Reaction Time. To adjust for the Type-1 error rate, the alpha level was adjusted to a Bonferroni-corrected *p*-value of *p* < 0.0125. Partial-eta squared (*η^2^*) was calculated as a measure of effect size, with 0.01 constituting a small effect, 0.06 a medium effect, and 0.14 a large effect (35).

The percentages of athletes in each group (No ADHD or LD, LD only, ADHD+LD, ADHD only) obtaining ImPACT composite scores using the following cutoffs were calculated: <25th, <16th, <10th, ≤ 5th, and ≤ 2nd percentiles. Chi-square analyses were then conducted comparing the percentage of athletes in the ADHD/LD groups (LD only, ADHD+LD, ADHD only) to the No ADHD or LD group (i.e., athlete control sample) separately for boys/young men and girls/young women. Odds ratio were calculated and Psi (*ψ*) was calculated as a measure of effect size, with 0.10 representing a small effect, 0.30 representing a medium effect, and 0.50 representing a large effect (35).

## 3 Results

For boys/young men, ANOVAs revealed statistically significant differences between groups (ADHD only, LD only, ADHD + LD, and No ADHD or LD) on ImPACT Verbal Memory [*F*(3, 117,447) = 1,043, *p* < 0.001, η^2^ = 0.03, small effect], Visual Memory [*F*(3, 117,447) = 913, *p* < 0.001, η^2^ = 0.02, small effect], Motor Speed [*F*(3, 117,447) = 3,261, *p* < 0.001, η^2^ = 0.08, medium effect] and Reaction Time scores [*F*(3, 117,447) = 799, *p* < 0.001, η^2^ = 0.02, small effect]. *Post hoc* analyses revealed that all groups differed from one another on each composite raw score (*p* < 0.001), with the No ADHD or LD group having the highest scores followed by ADHD Only, ADHD + LD, and LD Only groups (in that order, from highest to lowest scores).

For girls/young women, ANOVAs also revealed statistically significant differences between groups on ImPACT raw Verbal Memory [*F*(3, 75,396) = 1,210, *p* < 0.001, η^2^ = 0.05, small effect], Visual Memory [*F*(3, 75,396) = 871 *p* < 0.001, η^2^ = 0.03, small effect], Motor Speed [*F*(3, 75,396) = 3,139 *p* < 0.001, η^2^ = 0.11, medium-to-large effect], and Reaction Time [*F*(3, 75,396) = 950, *p* < 0.001, η^2^ = 0.04, small effect] scores. *Post hoc* analyses revealed that all groups differed from one another on each composite raw score (*p* < 0.001). The group differences followed the same pattern as boys/young men, with the No ADHD or LD group obtaining the highest scores followed by ADHD Only, ADHD + LD, and LD Only groups. See Table 2 for ImPACT composite score performance by group and sex.

**Table 2:** Means and standard deviations for ImPACT test performance by group and sex.

		*Verbal memory		*Visual memory		*Motor speed		*Reaction time	
		*M*	*SD*	*M*	*SD*	*M*	*SD*	*M*	*SD*
Males	**LD only	80.84	11.04	70.99	13.82	30.91	7.49	0.68	0.12
	**ADHD + LD	81.60	11.08	71.46	13.74	33.11	7.58	0.67	0.12
	**ADHD only	83.49	10.59	73.68	13.28	35.23	7.32	0.65	0.11
	**No ADHD or LD	85.61	10.23	76.48	12.63	36.59	6.96	0.64	0.09
Females	**LD only	82.78	11.08	69.86	13.78	31.85	7.08	0.69	0.12
	**ADHD + LD	83.77	11.04	70.36	13.98	33.96	7.24	0.68	0.11
	**ADHD only	85.69	10.5	72.15	13.64	36.02	6.95	0.66	0.11
	**No ADHD or LD	88.30	9.51	75.69	12.72	37.89	6.58	0.64	0.09

*ANOVAs: all *p* < 0.001; **Post hoc: No ADHD or LD > ADHD Only > ADHD + LD > LD Only, for all measures. ADHD, Attention-deficit/hyperactivity disorder; LD, Learning disability.

Regarding frequency of low scores across various percentile cutoffs (i.e., <25, <16, <10%, ≤ 5% and ≤ 2%), compared to student-athletes in the No ADHD or LD group, student-athletes in all ADHD/LD groups (LD only, ADHD only, and ADHD + LD) were significantly more likely to obtain at least one composite score below each percentile cutoff (*p* < 0.001). This finding held for both boys/young men and girls/young women. Odds ratios and effect sizes are presented in Table 3.

**Table 3:** Chi-square analysis of likelihood to fall below percentile cutoffs: all groups vs. control, stratified by sex.

	No ADHD or LD (control sample)	LD only		ADHD + LD		ADHD only	
Cutoff	% Below	% Below	OR/Phi	% Below	OR/Phi	% Below	OR/Phi
<25th %ile: Male	53.3	75.8	2.74/0.23	69.7	2.01/0.17	58.5	1.23/0.05
<25th %ile: Female	54.9	78.9	3.08/0.25	73.1	2.24/0.18	64.1	1.47/0.09
<16th %ile: Male	37.8	62.4	2.72/0.24	55.2	2.02/0.17	43.7	1.28/0.06
<16th %ile: Female	38.4	65.3	3.02/0.27	58.7	2.28/0.20	47.5	1.45/0.09
<10th %ile: Male	25.0	49.1	2.90/0.24	41.9	2.16/0.18	31.0	1.35/0.07
<10th %ile: Female	25.4	51.6	3.14/0.27	45.0	2.41/0.20	34.3	1.54/0.10
≤ 5th %ile: Male	15.6	36.6	3.13/0.23	30.1	2.33/0.17	20.5	1.40/0.06
≤ 5th %ile: Female	15.8	38.7	3.36/0.26	32.2	2.53/0.19	22.9	1.58/0.09
≤ 2nd %ile: Male	7.7	23.3	3.64/0.21	18.1	2.65/0.15	11.6	1.58/0.06
≤ 2nd %ile: Female	8.1	25.7	3.94/0.24	20.5	2.95/0.18	13.4	1.77/0.09

% Below reflects the percentage of cases falling below cutoffs for that percentile. *OR and Phi reflect comparison of each individual group to the No ADHD or LD group (control sample). **all *p* < 0.001. ADHD, Attention-deficit/hyperactivity disorder; LD, Learning disability.

Percentages for individuals obtaining scores below the various percentile cutoffs (i.e., <25, <16, <10%, ≤ 5% and ≤ 2%) are presented in Tables 4–7. For both high school and collegiate student-athletes who do not have ADHD or LD (athlete controls), obtaining one or more scores below the 10th percentile occurred in 24–26% of participants, whereas obtaining two or more scores below the 10th percentile occurred in just 6–7% of the sample (Table 4). However, for student-athletes with ADHD (Table 5), obtaining one or more scores below the 10th percentile was common (i.e., 27–35%), but obtaining two or more scores below the 10th percentile was uncommon (i.e., 8–12%). For student-athletes with LD (Table 6), although obtaining two or more scores below the 10th percentile was common (i.e., 20–24%), obtaining three or more scores below the 10th percentile was uncommon, occurring in 6–8% of the sample. Finally, for student-athletes with ADHD and LD (Table 7), obtaining two or more scores below the 10th percentile was relatively common (i.e., 15–19%), whereas obtaining three or more scores below the 10th percentile was uncommon (i.e., 5–6%). This pattern was consistent across age and sex groups, at each of the five percentile cutoffs.

**Table 4:** Base rates of low ImPACT scores for student-athletes in the control sample (without ADHD or LD) stratified by age and sex.

Number of scores below cutoff	Girls, ages 13–18 (*n* = 21,140)		Boys, ages 13–18 (*n* = 19,001)		Women, ages 19–22 (*n* = 3,669)		Men, ages 19–22 (*n* = 3,874)	
	%	C%	%	C%	%	C%	%	C%
<25th %ile								
4	2.2	2.2	1.9	1.9	1.7	1.7	2.3	2.3
3	6.9	9.1	6.7	8.7	6.4	8.1	7.1	9.4
2	16.4	25.6	15.5	24.2	16.1	24.2	16.3	25.7
1	29.4	55.0	29.0	53.2	30.1	54.3	28.6	54.3
0	45.0	100	46.8	100	45.7	100	45.7	100
<16th %ile								
4	0.7	0.7	0.7	0.7	0.7	0.7	0.9	0.9
3	3.2	3.9	3.1	3.7	2.4	3.1	2.8	3.7
2	9.9	13.8	9.3	13.0	9.4	12.5	10.7	14.4
1	25.0	38.8	24.7	37.7	24.0	36.5	24.0	38.3
0	61.2	100	62.3	100	63.5	100	61.7	100
<10th %ile								
4	0.2	0.2	0.2	0.2	0.2	0.2	0.3	0.3
3	1.3	1.5	1.4	1.6	1.0	1.2	1.4	1.7
2	5.2	6.7	5.1	6.6	4.9	6.1	5.5	7.2
1	18.9	25.6	18.4	25.0	17.6	23.7	17.9	25.1
0	74.4	100	75.0	100	76.3	100	74.9	100
≤ 5th %ile								
4	0.1	0.1	0.1	0.1	–	–	0.1	0.1
3	0.5	0.6	0.5	0.6	0.5	0.5	0.5	0.6
2	2.5	3.1	2.5	3.0	2.2	2.7	2.9	3.5
1	13.0	16.1	12.5	15.5	12.4	15.1	12.3	15.9
0	83.9	100	84.5	100	84.9	100	84.1	100
≤ 2nd %ile								
4	–	–	–	–	–	–	–	–
3	0.1	0.1	0.1	0.1	0.1	0.1	0.1	0.1
2	0.9	1.0	0.9	1.0	0.9	1.0	1.0	1.1
1	7.0	8.0	6.6	7.7	7.5	8.5	6.9	8.0
0	92.0	100	92.3	100	91.5	100	92.0	100

C%, Cumulative percentage.

**Table 5:** Age base rates of low ImPACT scores for student-athletes with ADHD only stratified by age and sex.

Number of scores below cutoff	Girls, ages 13–18 (*n* = 11,677)		Boys, ages 13–18 (*n* = 29,093)		Women, ages 19–22 (*n* = 1,491)		Men, ages 19–22 (*n* = 2,954)	
	%	C%	%	C%	%	C%	%	C%
<25th %ile								
4	4.1	4.1	2.8	2.8	3.0	3.0	2.3	2.3
3	10.9	15.0	8.8	11.6	7.5	10.5	6.7	9.0
2	20.8	35.8	18.2	29.8	16.1	26.6	17.2	26.2
1	29.5	65.3	29.0	58.8	29.2	55.8	29.0	55.2
0	34.7	100	41.2	100	44.2	100	44.8	100
<16th %ile								
4	1.4	1.4	1.2	1.2	0.9	0.9	1.0	1.0
3	5.7	7.1	4.3	5.5	3.8	4.7	3.5	4.5
2	14.0	21.1	11.7	17.2	9.7	14.4	10.2	14.7
1	27.5	48.6	26.8	44.0	24.6	39.3	25.8	40.5
0	51.4	100	56.0	100	60.7	100	59.5	100
<10th %ile								
4	0.6	0.6	0.5	0.5	0.2	0.2	0.4	0.4
3	2.4	3.0	1.9	2.4	2.0	2.2	1.6	2.0
2	8.5	11.6	7.3	9.7	6.4	8.6	5.7	7.7
1	23.7	35.3	21.6	31.3	18.6	27.2	19.1	26.8
0	64.7	100	68.7	100	72.8	100	73.2	100
≤ 5th %ile								
4	0.3	0.3	0.2	0.2	0.1	0.1	0.2	0.2
3	1.0	1.3	0.8	1.0	0.7	0.8	0.6	0.8
2	4.8	6.0	4.0	5.0	3.3	4.1	2.9	3.7
1	17.4	23.4	15.9	20.9	15.1	19.2	13.5	17.2
0	76.6	100	79.1	100	80.8	100	82.8	100
≤ 2nd %ile								
4	0.1	0.1	–	–	–	–	–	–
3	0.4	0.5	0.3	0.3	0.1	0.1	0.3	0.3
2	2.1	2.6	1.6	1.9	1.9	2.0	1.2	1.5
1	11.1	13.7	9.9	11.8	9.8	11.8	8.1	9.6
0	86.3	100	88.2	86.2	88.2	100	90.4	100

ADHD, Attention-deficit/hyperactivity disorder; C%, Cumulative percentage.

**Table 6:** Base rates of low ImPACT scores for student-athletes with LD only stratified by age and sex.

Number of scores below cutoff	Girls, ages 13–18 (*n* = 19s,361)		Boys, ages 13–18 (*n* = 29,036)		Women, ages 19–22 (*n* = 2,190)		Men, ages 19–22 (*n* = 3,636)	
	%	C%	%	C%	%	C%	%	C%
<25th %ile								
4	9.2	9.2	6.9	6.9	7.4	7.4	7.9	7.9
3	18.4	27.6	16.7	23.6	15.7	23.1	15.8	23.7
2	26.2	53.8	25.2	48.8	23.6	46.7	24.1	47.8
1	25.8	79.6	27.4	76.2	25.8	72.5	24.9	72.7
0	20.4	100	23.8	100	27.5	100	27.3	100
<16th %ile								
4	4.3	4.3	3.1	3.1	2.9	2.9	3.1	3.1
3	11.3	15.6	9.9	13.0	8.8	11.7	9.9	13.0
2	21.4	37.0	20.0	33.0	18.6	30.3	18.9	31.9
1	29.2	66.2	29.7	62.7	27.4	57.7	27.8	59.7
0	33.8	100	37.3	100	42.3	100	40.3	100
<10th %ile								
4	1.8	1.8	1.5	1.5	1.2	1.2	1.4	1.4
3	6.4	8.2	5.3	6.8	5.1	6.3	5.5	6.9
2	15.5	23.7	14.7	21.5	14.1	20.4	13.8	20.7
1	28.6	52.3	28.0	49.5	25.3	45.7	25.5	46.2
0	47.7	100	50.5	100	54.3	100	53.8	100
≤ 5th %ile								
4	0.8	0.8	0.5	0.5	0.7	0.7	0.9	0.9
3	3.3	4.1	3.0	3.5	2.6	3.3	2.2	3.1
2	10.2	14.3	9.2	12.7	9.4	12.7	8.9	12.0
1	25.0	39.3	24.2	36.9	21.2	33.9	21.7	33.7
0	60.7	100	63.1	100	66.1	100	66.3	100
≤ 2nd %ile								
4	0.3	0.3	0.2	0.2	0.3	0.3	0.3	0.3
3	1.3	1.6	1.1	1.3	1.1	1.4	0.9	1.2
2	5.5	7.1	4.9	6.2	5.4	6.8	4.3	5.5
1	18.9	26.0	17.4	23.6	16.6	23.4	15.8	21.3
0	74.0	100	76.4	100	76.6	100	78.7	100

C%, Cumulative percentage; LD, Learning disability.

**Table 7:** Base rates of low ImPACT scores for student-athletes with ADHD and LD stratified by age and sex.

Number of scores below cutoff	Girls, ages 13–18 (*n* = 13,788)		Boys, ages 13–18 (*n* = 26,270)		Women, ages 19–22 (*n* = 2,084)		Men, ages 19–22 (*n* = 3,595)	
	%	C%	%	C%	%	C%	%	C%
<25th %ile								
4	7.3	7.3	5.6	5.6	5.7	5.7	6.0	6.0
3	16.0	23.3	13.8	19.4	12.3	18.0	12.1	18.1
2	23.7	47.0	22.3	41.7	22.0	40.0	20.3	38.4
1	26.9	73.9	28.4	70.1	28.5	68.5	27.6	66.0
0	26.1	100	29.9	100	31.5	100	34.0	100
<16th %ile								
4	3.3	3.3	2.6	2.6	2.3	2.3	2.2	2.2
3	9.1	12.4	7.9	10.5	6.0	8.3	7.0	9.2
2	18.6	31.0	16.8	27.3	15.9	24.2	15.0	24.2
1	28.6	59.6	28.4	55.7	28.1	52.3	27.9	52.1
0	40.4	100	44.3	100	47.7	100.0	47.9	100
<10th %ile								
4	1.3	1.3	1.1	1.1	1.2	1.2	0.9	0.9
3	4.9	6.2	4.4	5.5	3.4	4.6	3.7	4.6
2	13.1	19.3	11.5	17.0	10.9	15.5	10.8	15.4
1	26.4	45.7	25.2	42.2	25.0	40.5	23.4	38.8
0	54.3	100	57.8	100	59.5	100.0	61.2	100
≤ 5th %ile								
4	0.6	0.6	0.4	0.4	0.4	0.4	0.4	0.4
3	2.4	3.0	2.0	2.4	2.0	2.4	1.6	2.0
2	8.3	11.3	7.2	9.6	6.6	9.0	6.8	8.8
1	21.4	32.7	20.7	30.3	19.6	28.6	18.8	27.6
0	67.3	100	69.7	100	71.4	100	72.4	100
≤ 2nd %ile								
4	0.2	0.2	0.1	0.1	0.1	0.1	0.1	0.1
3	0.9	1.1	0.7	0.8	0.8	0.9	0.5	0.6
2	4.2	5.3	3.5	4.3	3.6	4.5	3.5	4.1
1	15.6	20.9	14.0	18.3	13.5	18.0	11.9	16.0
0	79.1	100	81.7	100	82.0	100	84.0	100

ADHD, Attention-deficit/hyperactivity disorder; C%, Cumulative percentage; LD, Learning disability.

## 4 Discussion

**Figure 1:**
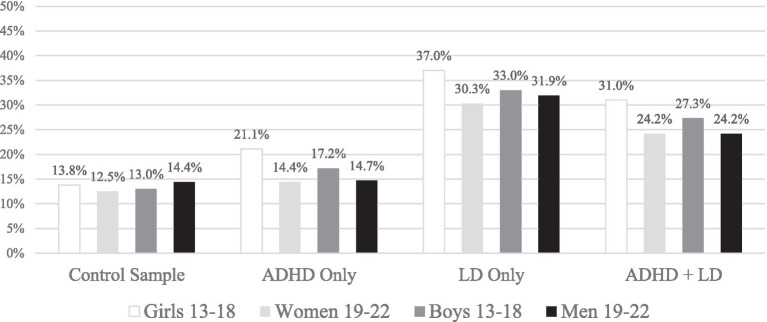
Percentages of student athletes with 2 or more ImPACT scores less than the 16th percentile (one standard deviation), stratified by age group and gender. Student athletes were included if they had valid scores on ImPACT. These percentages would be higher if those with invalid scores were included. ADHD, Attention-deficit/hyperactivity disorder; LD, Learning disability.

This study examined the frequency of low scores on preseason baseline ImPACT testing among student-athletes with ADHD, with or without co-occurring LD, or with LD only. The ImPACT composite scores in the current sample were quite similar to a previous study including samples of high school and college athletes with ADHD and/or LD (24). The application of multivariate base rate information in the current study is important when interpreting results across the entire ImPACT battery (i.e., interpreting performance as a whole, considering all four composites together). The multivariate base rates calculated in the current study show that, for the most part, uninjured student athletes with ADHD, with LD, or with both ADHD and LD are more likely than student athletes without these neurodevelopmental conditions to obtain a given number of low ImPACT composite scores. Specifically, around 13 to 14% of student-athletes ages 13–22 without ADHD and/or LD (the control sample) obtain two or more scores more than 1 standard deviation below the normative mean (i.e., below the 16th percentile). This was also true for student-athletes ages 19–22 with ADHD only (i.e., 14–15%, see Figure 1). However, a greater proportion (17–21%) of younger student-athletes (e.g., 13–18) with ADHD only, of student-athletes ages 13–22 with LD only (30–37%), and of student-athletes ages 13–22 with both ADHD and LD (24–31%) obtained two or more scores below the 16th percentile (Figure 1).

Clinicians and researchers alike can consider such base rate information when interpreting and considering what constitutes “normal” performance across the ImPACT battery. For instance, clinicians interpreting post-concussion ImPACT test scores are encouraged to consider that an uninjured student-athlete with *LD or comorbid ADHD/LD* would have a relatively high likelihood of obtaining two or more low scores. Therefore, having three low scores may be more likely indicative of cognitive deficit following concussion among some student-athletes with these neurodevelopmental conditions (see Table 8).

**Table 8:** Quick reference guide: percentages of student athletes with low (shaded) and uncommon (bolded) patterns of low scores.

	ADHD only	LD only	ADHD + LD	No ADHD/LD (control sample)
Girls and women				
Girls, 13–18	*n* = 11,667	*n* = 19,361	*n* = 13,788	*n* = 21,140
3+ <25th %ile	15.0%	27.6%	23.3%	**9.1%**
3+ <16th %ile	**7.1%**	15.6%	12.4%	**3.9%**
2+ <16th %ile	21.1%	37.0%	31.0%	13.8%
3+ <10th %ile	**3.0%**	**8.2%**	**6.2%**	**1.5%**
2+ <10th %ile	11.6%	23.7%	19.3%	**6.7%**
Women, 19–22	*n* = 1,491	*n* = 2,190	*n* = 2,084	*n* = 3,669
3+ <25th %ile	10.5%	23.1%	18.0%	**8.1%**
3+ <16th %ile	**4.7%**	11.7%	**8.3%**	**3.1%**
2+ <16th %ile	14.4%	30.3%	24.2%	12.5%
3+ <10th %ile	**2.2%**	**6.3%**	**4.6%**	**1.2%**
2+ <10th %ile	**8.6%**	20.4%	15.5%	**6.1%**
Boys and men				
Boys, 13–18	*n* = 29,093	*n* = 29,036	*n* = 26,270	*n* = 19,001
3+ <25th %ile	11.6%	23.6%	19.4%	**8.7%**
3+ <16th %ile	**5.5%**	13.0%	10.5%	**3.7%**
2+ <16th %ile	17.2%	33.0%	27.3%	13.0%
3+ <10th %ile	**2.4%**	**6.8%**	**5.5%**	**1.6%**
2+ <10th %ile	**9.7%**	21.5%	17.0%	**6.6%**
Men, 19–22	*n* = 2,954	*n* = 3,636	*n* = 3,595	*n* = 3,874
3+ <25th %ile	**9.0%**	23.7%	18.1%	**9.4%**
3+ <16th %ile	**4.5%**	13.0%	**9.2%**	**3.7%**
2+ <16th %ile	14.7%	31.9%	24.2%	14.4%
3+ <10th %ile	**2.0%**	**6.9%**	**4.6%**	**1.7%**
2+ <10th %ile	**7.7%**	20.7%	15.4%	**7.2%**

In column one, the number of low scores below each percentile (%) cutoff, such as having 3 or more scores below the 25th percentile, are represented as each pattern of low scores. Boxes that are shaded represent low patterns of performance, occurring in fewer than 16% of the sample, representing a pattern that is below one standard deviation from the mean. Bolded percentages are uncommon patterns of low scores, occurring in fewer than 10% of the sample. ADHD, Attention-deficit/hyperactivity disorder; LD, Learning disability.

Obtaining a single low score (or more) was common in the control sample, especially for conventional cutoffs for defining low scores, such as those below the 25th, 16th, and 10th percentiles (Table 4). However, obtaining two low composite scores occurs infrequently in the control sample (Table 4, Figure 1, and Table 8). This aligns with multiple other studies. In a sample of high school and college athletes (without ADHD and/or LD) using ImPACT, falling below percentile-based cut-offs on two of the ImPACT composite scores was uncommon (8). Similarly, in the ImPACT Pediatric normative sample, it is uncommon for children ages 5–11 (without ADHD and/or LD) to have two low ImPACT Pediatric factor/composite scores (36). Utilizing the ImPACT Quick Test (a brief neurocognitive screening measure) in the normative sample of individuals ages 12–60 (without ADHD and/or LD), having two low factor/composite scores is uncommon (37). Finally, in a sample of adolescents (ages 13–19) completing ImPACT, scoring below cutoffs on three of the ImPACT scores was uncommon in participants who identify as Black or come from lower socioeconomic backgrounds, as compared to participants who identify as White or come from middle-to-high socioeconomic backgrounds (37). We extend this body of work by creating multivariate base rate tables for adolescent and young adult student athletes who have neurodevelopmental disorders. In Table 8, we provide a quick reference guide for clinicians—and this can be used to more precisely interpret patterns of low scores on ImPACT in student athletes who have (or do not have) neurodevelopmental disorders.

### 4.1 Clinical implications for assessing sport-related concussion

These findings have implications for clinicians, physicians, and sports medicine professionals utilizing neurocognitive test measures for the assessment and management of sports-related concussion. Approximately 10–16% of children and adolescents (14–16) are diagnosed with ADHD and 9.7% are diagnosed with LD (38). Therefore, the current results might assist interpretation of ImPACT test performance for as many as 1 in 10 high school and collegiate athletes who may have ADHD or LD. It is important to note that in the absence of available baseline data for athletes with ADHD or LD, post-injury test scores are commonly compared to age-and sex-based normative data to assess for a possible deficit in functioning associated with a concussion. However, per the current findings and several prior studies, student-athletes with ADHD or LD are more likely to obtain low scores than those without these neurodevelopmental conditions. Thus, normative comparisons may misclassify student-athletes with ADHD and/or LD as scoring below expectation, when, in fact, their scores may be broadly normal compared to individuals who have neurodevelopmental conditions. Given that individuals with ADHD and/or LD are more likely to score below cutoffs that were generated using normative samples of individuals mostly or entirely without ADHD and/or LD, clinicians may consider a more targeted and individualized approach to interpreting neurocognitive test data in these student-athletes, such as provided here. Notably, athletes with ADHD and/or LD were excluded from the ImPACT normative samples (27). Thus, the multivariate base rates of low scores for student-athletes with ADHD and/or LD reported in this study are recommended to enhance the accuracy of interpreting ImPACT performance among athletes with these neurodevelopmental conditions.

### 4.2 Future research

Additional research may help establish how different levels of severity of ADHD and/or LD can affect neurocognitive test performance in student-athletes. Overall, individuals with LD consistently performed the worst on ImPACT composite scores compared to those with ADHD, both ADHD and LD, and those without either condition. This was a somewhat surprising finding because it was hypothesized that the comorbid ADHD + LD group would be the most likely to obtain low scores, given the combined presence of two neurodevelopmental disorders that have both been independently linked to lower neurocognitive test scores (25). One possible explanation for this result could be that those included in the LD only group may be experiencing more severe forms of LD than those in the ADHD + LD group. It is also possible that student-athletes with comorbid ADHD are receiving pharmacotherapy for their condition that may affect neurocognitive test performances. Future research examining ADHD and/or LD severity and medication use could clarify differences in cognitive test performance between student-athletes with ADHD and LD. Finally, while prior history of concussion was not included as an additional variable in the analyses, several studies have not revealed a meaningful association between prior history of concussions and baseline ImPACT test scores (39–43). That said, future multivariate base rate research could examine whether base rates of low scores vary in association with 0, 1, or 2+ previous concussions, as a way confirm the lack of association between concussion history and baseline ImPACT scores—but such stratifications were beyond the scope of the current study.

### 4.3 Limitations

There are limitations to the current study. First, the presence of neurodevelopmental disorders (ADHD and/or LD) were obtained through athlete self-report data. It is possible that some cases were inaccurately classified as having these conditions. We were not able to confirm diagnostic status. It is notable, however, that in a large-scale study of adolescent student athletes who underwent baseline testing twice, they reported both their concussion history and their neurodevelopmental history (e.g., whether they had a diagnosis of ADHD or LD) very consistently across the two baseline evaluations (44). Second, ADHD and/or LD were binary self-report variables (yes or no), whereas symptoms of ADHD and LD fall across a range of severity, especially for males (45). Further, diagnostically ADHD and LD are characterized as either “mild,” “moderate,” or “severe,” with regard to the number of symptoms present and the degree and range of functional impairment experienced (17). It was not possible to weigh or evaluate the influence of ADHD or LD symptom severity or functional impairment in this study, which represents an important future direction. It was also not possible to classify participants based on ADHD or LD subtypes. Diagnostically, ADHD is characterized by three subtypes (i.e., predominantly inattentive presentation, predominantly hyperactive/impulsive presentation, or combined presentation) and LD is characterized by the specific area of academic impairment (i.e., with impairment in reading, with impairment in written expression, and/or with impairment in mathematics) (17). It is possible that individuals might differ on ImPACT performance and number of low scores obtained based on ADHD subtype and/or the specific form or forms of LD.

Third, this study did not consider repetitive head impact exposure associated with playing different sports, and whether that might be associated with the base rates of low scores. Recent research, however, suggest that there are not meaningful differences in ImPACT composite scores in adolescents that are associated with them playing non-contact, contact, or collision sports (46, 47). Fourth, although participants with recent concussions (i.e., within the last 6 months) were excluded from analysis, participants with prior remote concussions were included in this study, but concussion history was not considered in the multivariate base rates calculation. However, participants with prior concussions were represented in all groups (i.e., control participants, as well as participants with ADHD, LD, and ADHD + LD). And, as noted above, past studies have not revealed a meaningful association between prior history of concussions and baseline ImPACT test scores (39–43).

Fifth, invalid baselines were removed prior to multivariate base rate analyses, because these scores would be flagged as invalid and not recommended for interpretation in clinical practice. It is possible, of course, that some of the “invalid” scores are actually “valid” (but simply low)—and it has been established that youth with neurodevelopmental disorders are more likely to obtain scores labelled as “invalid” on the ImPACT score interpretation printout (48). Including youth with invalid scores would, by definition, increase the base rates of low scores in the current study for the total sample and all subgroups. However, this would make the results somewhat less translatable to clinical practice, in which those scores would traditionally not be interpreted (or be interpreted cautiously). As such, we thought their inclusion would have decreased the clinical usefulness and translational value of the base rates prepared for this study.

Finally, additional variables that may be related to ImPACT test scores, such as race, socioeconomic status (SES), stereotype threat, ADHD medication status, and fatigue, were neither measured nor included. Researchers have shown that race and SES are related to obtaining low scores on ImPACT (37), and that Black children (14) and children from families of lower SES (49) are more likely to be diagnosed with ADHD. Consideration of broader social and structural factors as they relate to concussion assessment and management represent a major priority for the field moving forward.

### 4.4 Conclusion

In summary, uninjured student-athletes with ADHD and/or LD were more likely to obtain low ImPACT test scores than those without ADHD or LD. Moreover, those with LD were most likely to have two or more low scores. Multivariate base rates highlight that it was common for student-athletes with ADHD and/or LD to obtain low scores in the absence of concussive injury, and provide reference values for the typical rate of obtaining low scores on ImPACT. This study made use of a large ImPACT database, with strong generalizability to the normative reference group used in clinical practice. Having information relating to the base rates of low scores enhances the interpretation of ImPACT results among the broader population of student-athletes with and without neurodevelopmental disorders.

## Data Availability

The datasets presented in this article are not readily available because they were provided to the authors through a cooperative research agreement with ImPACT Applications, Inc. The statistical analyses and outputs are available to qualified researchers, for research purposes. Requests to access the datasets should be directed to pschatz@sju.edu.
